# Distribution and diversity of the haemoglobin–haptoglobin iron-acquisition systems in pathogenic and non-pathogenic *Neisseria*

**DOI:** 10.1099/mic.0.068874-0

**Published:** 2013-09

**Authors:** Odile B. Harrison, Julia S. Bennett, Jeremy P. Derrick, Martin C. J. Maiden, Christopher D. Bayliss

**Affiliations:** 1 Department of Zoology, University of Oxford, South Parks Road, Oxford OX1 3SY, UK; 2 Department of Genetics, University of Leicester, Leicester LE1 7RH, UK; 3 Faculty of Life Sciences, University of Manchester, Manchester M13 9PT, UK

## Abstract

A new generation of vaccines containing multiple protein components that aim to provide broad protection against serogroup B meningococci has been developed. One candidate, 4CMenB (4 Component MenB), has been approved by the European Medicines Agency, but is predicted to provide at most 70–80 % strain coverage; hence there is a need for second-generation vaccines that achieve higher levels of coverage. Prior knowledge of the diversity of potential protein vaccine components is a key step in vaccine design. A number of iron import systems have been targeted in meningococcal vaccine development, including the HmbR and HpuAB outer-membrane proteins, which mediate the utilization of haemoglobin or haemoglobin–haptoglobin complexes as iron sources. While the genetic diversity of HmbR has been described, little is known of the diversity of HpuAB. Using whole genome sequences deposited in a Bacterial Isolate Genome Sequence Database (BIGSDB), the prevalence and diversity of HpuAB among *Neisseria* were investigated. HpuAB was widely present in a range of *Neisseria* species whereas HmbR was mainly limited to the pathogenic species *Neisseria meningitidis* and *Neisseria gonorrhoeae*. Patterns of sequence variation in sequences from HpuAB proteins were suggestive of recombination and diversifying selection consistent with strong immune selection. HpuAB was subject to repeat-mediated phase variation in pathogenic *Neisseria* and the closely related non-pathogenic *Neisseria* species *Neisseria lactamica* and *Neisseria polysaccharea* but not in the majority of other commensal *Neisseria* species. These findings are consistent with HpuAB being subject to frequent genetic transfer potentially limiting the efficacy of this receptor as a vaccine candidate.

## Introduction

*Neisseria meningitidis*, the meningococcus, continues to cause meningitis and septicaemia worldwide with a high mortality rate and frequent debilitating sequelae. Meningococci are, however, normally commensal residents of the oropharynx of a high percentage (10–30 %) of the human population and can persist asymptomatically in the carrier state for periods of months to years (Caugant & Maiden, 2009). The closely related gonococcus, *Neisseria gonorrhoeae*, on the other hand, causes a widespread sexually transmitted disease, involving colonization and invasion of the mucosal surfaces of the genital tract with occasional ascension that leads to serious sequelae such as ectopic pregnancy, sterility and disseminated infection (Criss & Seifert, 2012). The gonococcus can also persist in asymptomatic carriers. Other *Neisseria* species are also localized to the upper respiratory tracts of humans, and related organisms occur in other vertebrates. Some of these have occasionally been associated with disease but they are essentially non-pathogenic. For example, *Neisseria lactamica* is a commensal frequently isolated in the upper respiratory tracts of young children but is very rarely associated with pathology (Bennett *et al.*, 2012).

The most effective *N. meningitidis* vaccines are composed of capsular polysaccharide antigens conjugated to a carrier protein and these have been successfully used to induce protective immunity against *N. meningitidis* serogroups A, C, W and Y (Pollard, 2004). No such vaccine is available against serogroup B *N. meningitidis* due to similarities between the serogroup B polysaccharide and human glycoprotein structures, and no vaccines targeting *N. gonorrhoeae* isolates are available. Protein-based vaccines have been developed to protect against *N. meningitidis* strains expressing serogroup B capsules and these include a range of formulations such as 4CMenB (4 Component MenB, Bexsero®; Novartis Vaccines and Diagnostics) (Serruto *et al.*, 2012) and rLP2086 (Pfizer) (Richmond *et al.*, 2012); however, due to incomplete vaccine coverage, there is a requirement for second-generation vaccines which achieve higher levels of coverage by the inclusion of more variants of existing components and/or the inclusion of additional components (Lucidarme *et al.*, 2010; Vogel *et al.*, 2013).

Surface receptors that mediate iron acquisition are attractive potential vaccine components as they are essential for growth in the iron-limited conditions prevailing in host tissues. Meningococci express five such receptors: (i) the transferrin binding proteins TbpA and TbpB; (ii) the lactoferrin binding proteins LbpA and LbpB; (iii) the FrpB/FetA transporter; (iv) the haemoglobin receptor HmbR; and (v) the haptoglobin–haemoglobin receptors HpuAB (Perkins-Balding *et al.*, 2004; Saleem *et al.*, 2013). Transferrin and lactoferrin receptors are outer-membrane proteins (OMPs) involved in acquisition of iron from transferrin and lactoferrin and are almost universally present in meningococci and gonococci. Infection of human volunteers with gonococcal mutants has indicated a requirement for these receptors for efficient colonization of the male urethra (Cornelissen *et al.*, 1998) and the recent publication of the TbpA and TbpB protein structures is prompting some renewed interest in these proteins as vaccine candidates (Calmettes *et al.*, 2012; Noinaj *et al.*, 2012b). HmbR is a transmembrane protein with specificity for haemoglobin, whilst the bipartite receptor HpuAB can bind both haemoglobin and haptoglobin–haemoglobin complexes (Lewis *et al.*, 1999; Stojiljkovic *et al.*, 1995).

The acquisition of iron from haemoglobin, lactoferrin and transferrin in *Neisseria* is mediated by receptors composed of two distinct surface-exposed OMPs with very different properties and specificities (Cornelissen & Hollander, 2011; Noinaj *et al.*, 2012a). The first protein is TonB-dependent and functions as a pore through which the iron or haem is directly transported. The second is a fully surface-exposed lipoprotein which is thought to act as an accessory protein enhancing the specificity and strength of ligand binding. In the HpuAB system, HpuB is the TonB-dependent OMP acting as a pore with HpuA being the fully surface-exposed lipoprotein.

HmbR and HpuAB receptors are variably present in *Neisseria* isolates with four combinations of the receptors observed: (i) both receptors; (ii) HmbR only; (iii) HpuAB only; and (iv) neither receptor present (Tauseef *et al.*, 2011). Among meningococci associated with disease, there is a significant over-representation of the presence of HmbR and an under-representation of an HpuAB only phenotype. Further there is a high prevalence of both receptors in clonal complexes frequently associated with invasion relative to their carriage prevalence (Harrison *et al.*, 2009). Both of these receptors are subject to phase variation due to alterations in poly G repeat tracts present within the reading frame, with a high frequency of expression observed in meningococci associated with disease (Tauseef *et al.*, 2011). These findings suggest that Tbp and Lbp are required for colonization of mucosal surfaces but that the haemoglobin receptors may facilitate invasion and dissemination in the vascular system.

While the molecular evolution of the HmbR protein has been investigated in meningococci (Evans *et al.*, 2010), little is known about the diversity and selection pressures acting on the HpuAB OMPs and thus the suitability of this receptor as a vaccine candidate has not been explored. This study showed that HpuAB was present in a range of *Neisseria* species and that HmbR was mainly found in the pathogenic species *Neisseria meningitidis* and *Neisseria gonorrhoeae*. Two HpuAB families were evident: one present in non-pathogenic *Neisseria* species and having a non-phase variable *hpuA* gene and the other only observed in *N. meningitidis*, *N. gonorrhoeae*, *Neisseria lactamica* and *Neisseria polysaccharea*. Analysis of selection and recombination acting on HpuAB sequences revealed a number of residues were subject to immune selection and putatively located in surface-exposed regions of the protein. These findings were consistent with HpuAB being part of the accessory genome and subject to frequent exchange of allelic sequences possibly reducing the utility of this receptor as a vaccine antigen. Furthermore, the prevalence of these genes in the closely related non-pathogenic *Neisseria* population indicates that a vaccine targeting these antigens might have an effect on the commensal microbiota with implications for natural immunity.

## Methods

### 

#### Neisseria isolates.

A total of 218 isolates were investigated. These included the 107 *N. meningitidis* isolates employed in the evaluation of the MLST method (Maiden *et al.*, 1998) (Table S1, available in *Microbiology* Online), additional *N. meningitidis* carriage isolates with distinct serogroups (L, H, I and K) (Harrison *et al.*, 2013) along with *N. meningitidis* isolates obtained during a well-characterized disease outbreak in 1997 in Southampton, UK (Jolley *et al.*, 2012) and several serogroup B isolates for which whole genome sequences were available (Budroni *et al.*, 2011) (Table S1).

A total of 16 *N. gonorrhoeae* isolates, the genomes of which had been sequenced by the Broad Institute, were included (*Neisseria gonorrhoeae* group Sequencing Project, Broad Institute of Harvard and MIT (http://www.broadinstitute.org)), along with 81 commensal *Neisseria* obtained from a number of sources including: the Culture Collection of the University of Goteborg (CCUG); the American type Culture Collection (ATCC); and a study of asymptomatic carriers in Oxfordshire (Bennett *et al.*, 2005) (Table S1). Species definitions were those based on ribosomal multi locus sequence typing (rMLST) (Bennett *et al.*, 2012). Whole genome sequence data are available on the PubMLST website (www.pubmlst.org/neisseria).

#### Next generation sequencing and annotation of sequence data.

Sequencing, assembly, uploading and annotation of genomic sequences were performed as described previously (Bennett *et al.*, 2012). Briefly, genomic DNA was prepared following overnight growth on Columbia horse blood agar plates (Oxoid) using a Wizard Genomic DNA Purification kit (Promega). Pooled libraries of sheared genomic DNA were subject to paired end sequencing on an Illumina Genome Analyser II platform. Genome sequence data were assembled using velvet version 1.0.10 with optimal parameters determined by the velvetoptimizer.pl script within the software package (Zerbino, 2010) with the resultant contigs uploaded into a Bacterial Isolate Genome Sequence Database (BIGSDB) along with any available provenance data. Genome sequence data from other studies were uploaded directly to the database (Table S1).

Sequence definitions were generated for *hpuA*, *hpuB,* and *hmbR* genes in the sequence definition database and seeded with corresponding reference nucleotide sequences. Iterative searches of genome sequence data using progressively decreasing stringency settings by means of blastn or tblastx algorithms (Altschul *et al.*, 1997) identified *hpuA*, *hpuB* and *hmbR* genes which were then selected in the genomes enabling them to be extracted and exported for further analysis. Arbitrary allele numbers were assigned to each unique sequence for a given locus. Gene sequences were exported as XMFA files containing aligned sequence blocks and then converted to a fasta format for import into mega version 5.0 and Splitstree (Huson & Bryant, 2006; Tamura *et al.*, 2011). In addition, genetic diversity arising from the slipped-strand mispairing region encoded by the polyG tract was removed in *hpuA* sequences. All *hpuA* and *hpuB* sequences are available through the PubMLST database (www.pubmlst.org/neisseria). In addition to the common gene name, these loci are assigned a value-free nomenclature (NEIS1946 and NEIS1947) following on from the FAM18 genome annotation but using the prefix NEIS instead of NMC.

#### Analysis of gene sequences.

Isolates containing complete nucleotide sequences for both *hpuA* and *hpuB* were selected for phylogenetic analyses (*n* = 151). mega version 5.0 was used to calculate overall *p-*distances of *hpuA* and *hpuB* nucleotide sequences as well as *p-*distance values within Family A or B genes or within each specific region from the putative HpuB structural topological model. neighbournet trees were constructed using Splitstree version 4.10 (Huson & Bryant, 2006). The characterization of selection in the presence of recombination was carried out using the omegamap software package (Wilson & McVean, 2006), which employs a Bayesian approach to parameter estimation that is independent of phylogeny and was therefore less likely to falsely identify sites subject to diversifying selection in sequences displaying clear evidence of recombination. The signature of natural selection was detected using the *d_N_/d_S_* ratio and the signature of recombination was detected from the patterns of linkage disequilibrium. In the present study, three runs composed of 100 000 iterations and 100 000 burn-ins each were undertaken, compared to assess convergence and combined. Output from the omegamap runs was used to visualize possible selection acting on the sequence by means of a graph indicating the posterior probability of positive selection along the sequence and using the statistics package R version 2.15.1 (http://www.r-project.org).

#### Generation of a structural topological model for HpuB.

Amino acid sequence alignments of HpuA and HpuB with TbpB or TbpA respectively, for which crystal structures have been published, did not reveal significant sequence identities. A structural model for HpuB was generated using swiss-model (Arnold *et al.*, 2006) and the three-dimensional structure of the *Shigella dysenteriae* ShuA protein as a guide (Protein Data Bank PDB accession number 3FHH) (Cobessi *et al.*, 2010). Even using this model, the overall sequence identity was 16 %; several loop regions were excised from the final model as they had low homology with the equivalent sequences in ShuA and were too long to be modelled effectively.

## Results

### Distribution of haemoglobin receptor genes in pathogenic and non-pathogenic *Neisseria*

The majority (88 %) of the *N. meningitidis* isolates examined contained *hmbR* genes with 37 % having *hmbR* alone whilst only 12 % had *hpu* alone (Table 1). The *hmbR* only genotype was particularly noticeable among serogroup B meningococci belonging to clonal complexes ST-18, ST-32 and ST-41/44 (Tauseef *et al.*, 2011). All of the *N. gonorrhoeae* isolates contained *hpuAB* and *hmbR* genes, but the latter were all pseudogenes.

**Table 1.** Distribution of haemoglobin receptors in *Neisseria* species

Species*	Both	HpuAB only	HmbR only	Neither
*N. meningitidis* (*n* = 120)	62	14	44	0
*N. gonorrhoeae* (*n* = 16)	16‡	0	0	0
*N. polysaccharea* (*n* = 5)	2‡	3	0	0
*N. ‘bergeri’* (*n* = 1)	1	0	0	0
*N. lactamica* (*n* = 22)	0	22	0	0
*N. cinerea* (*n* = 7)	0	7	0	0
*N. subflava* (*n* = 16)	0	15	1	0
*N. mucosa* (*n* = 12)	0	10	2	0
*N. mucosa var. heidelbergensis*† (*n* = 4)	0	1	3	0
*N. (unknown)* (*n* = 2)	0	1	0	1
*N. bacilliformis* (*n* = 6)	0	0	0	6
*N. elongata variants* (*n* = 4)	0	0	1	3
*N. animalis* (*n* = 1)	0	0	0	1
*N. canis* (*n* = 1)	0	0	0	1
*N. dentiae* (*n* = 1)	0	0	0	1
*N. weaveri* (*n* = 1)	0	0	0	1
**Total**	**81**	**73**	**51**	**14**

* Species were defined based on rMLST designations (Bennett *et al.*, 2012).

† Also known as *N. oralis.*

‡ *hmbR* pseudogene.

Non-pathogenic *Neisseria* isolates exhibited a high prevalence of a *hpu* only genotype (71 %) with the majority (>80 %) of *N. lactamica*, *Neisseria cinerea*, *N. polysaccharea* and *Neisseria subflava* isolates containing this genotype (Table 1 and Table S1). In contrast, only 3 % (3/93) and 8 % (7/93) of the non-pathogenic isolates had genes encoding either receptors or a *hmbR* only genotype, respectively. The majority (*n* = 5) of *hmbR* only isolates among non-pathogenic *Neisseria* were present in *Neisseria mucosa*, which exhibited a split between a *hmbR* only genotype (31 %) (three of which in *Neisseria mucosa var. heidelbergensis,* which is also known as *Neisseria oralis* (Berger, 1971; Wolfgang *et al.*, 2013)) and a *hpuAB* only genotype (59 %) (Table S1). Many (14/93; 15 %) of the non-pathogenic species *Neisseria bacilliformis* and *Neisseria elongata* lacked both receptors (Table 1).

### Patterns of Hpu diversity

**Fig. 1.**
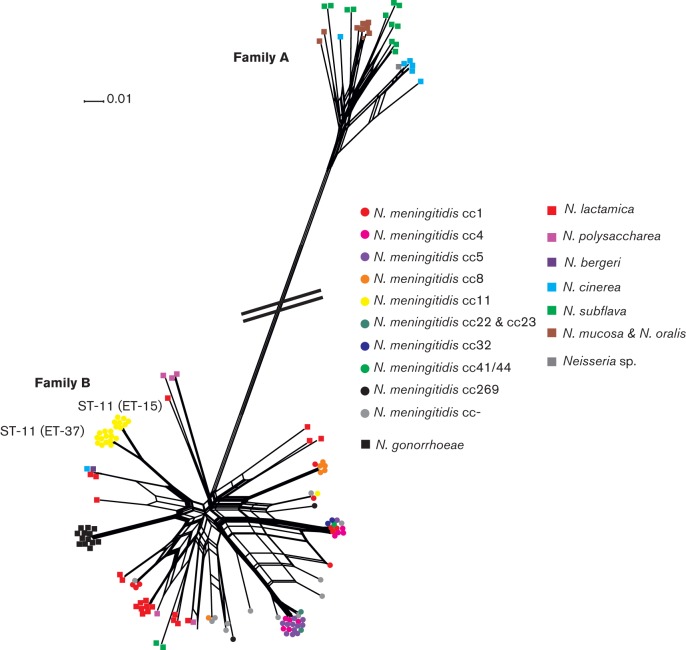
NEIGHBOURNET phylogeny created from *hpuA* nucleotide sequences from 151 *Neisseria* isolates. Families A and B are depicted. Circles denote *N. meningitidis* isolates and are colour coded by clonal complex. *N. gonorrhoeae* isolates are represented by black squares. Squares also depict the non-pathogenic *Neisseria* species which are colour coded. Scale bar represents 1% nucleotide substitutions per site with a fit index of 96%.

**Fig. 2.**
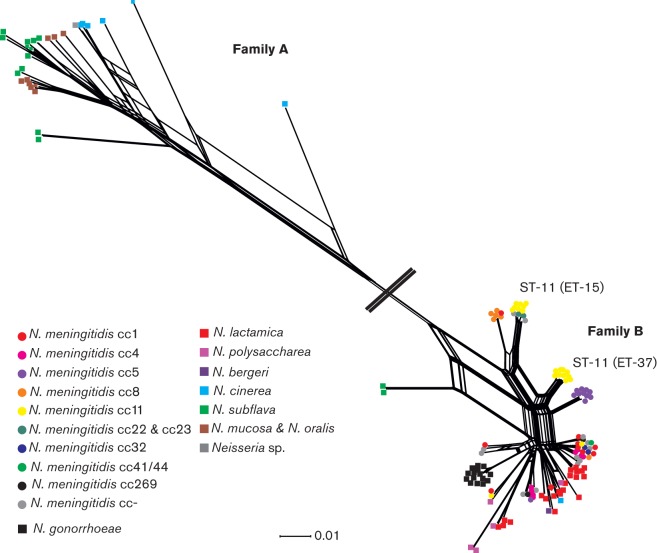
NEIGHBOURNET phylogeny created from *hpuB* nucleotide sequences from 151 *Neisseria* isolates. Families A and B are depicted. Circles denote *N. meningitidis* isolates and are colour coded by clonal complex. *N. gonorrhoeae* isolates are represented by black squares. Squares also depict the non-pathogenic *Neisseria* species which are colour coded. Scale bar represents 1% nucleotide substitutions per site with a fit index of 97%.

The nucleotide sequences for *hpuA* genes ranged in size from 909 to 1017 bp (303–339 amino acids) while *hpuB* sequences were substantially larger ranging in size from 2418 to 2439 bp (806–813 amino acid residues). Comparison of the *hpuAB* sequences and phylogenetic reconstructions indicated the presence of two divergent families of HpuAB receptor: Family A occurred in most of the non-pathogenic *Neisseria* species; while Family B was found in the pathogenic meningococcus and gonococcus and the closely related organisms *N. polysaccharea, Neisseria bergeri and N. lactamica* (Bennett *et al.*, 2012) (Figs 1 and 2, Table 2). However, *hpuA* and *hpuB* sequences belonging to *N. subflava* isolates CCUG 24841 and CCUG 24844 and *N. cinerea* CCUG 5746 also contained Family B sequences (Table S1).

**Table 2.** Sequence diversity of *hpuA* and *hpuB* in 151 *Neisseria* isolates

Parameter	*hpuA* (906–1017 bp)			*hpuB* (2415–2436 bp)		
All	Family A	Family B	All	Family A	Family B
Nucleotide sequences	151	31	120	151	31	120
Nucleotide alleles	68	26	42	73	26	59
Variable nucleotide sites	622	527	356	1001	923	426
*p-*distance	0.195	0.147	0.100	0.100	0.102	0.038
Amino acid alleles	61	22	40	130	26	51
Variable amino acid sites	224	184	149	290	257	135

Within Family A, sequences derived from different *Neisseria* species were present in multiple, divergent lineages and did not form distinct clusters. In Family B, *N. gonorrhoeae hpuA* and *hpuB* sequences formed distinct clusters whilst *hpuA* and *hpuB* sequences from *N. lactamica*, *N. polysaccharea* and *N. subflava* were more diverse (Figs 1 and 2).

The *hpuA* genes of the Family A isolates were similar in length with only four short indels among the sequences examined – two of which were found in only one isolate. The *hpuA* sequences of the Family B isolates were variable in length with a ~60 nt insertion present in some isolates and numerous indels of 3–6 nt. The *hpuB* sequences of both families exhibited high levels of sequence similarity to each other with few indels. Extension of the sequence analysis to flanking regions showed that Family A isolates diverged immediately after the termination codon of *hpuB*, comprising variations in the types and numbers of repetitive sequences, whereas the Family B isolates displayed high levels of similarity even in this intergenic region.

Family A sequences did not contain the poly G repeat tract in *hpuA*. Translation of HpuA was predicted to start from an identical sequence in all of these isolates (5′ATGAAAATCA). Analysis of the flanking sequences detected variation immediately upstream of this initiation codon. In addition, further upstream there was a gene encoding a hypothetical protein that was conserved among some of the non-pathogenic species and exhibited a low level of amino acid homology to NMB1971 of *N. meningitidis* strain MC58 (data not shown). By contrast, all of the Family B sequences contained a poly G repeat tract starting at nucleotide 57 after the ATG start codon with the exception of those from *N. subflava* isolates CCUG 24841 and CCUG 24844 and *N. cinerea* CCUG 5746. A second feature of the Family A isolates is that the *hpuA* coding sequences terminate 53–56 nt upstream of the initiation codon of *hpuB* whereas the gap is only ~33 nt in the Family B sequences. Thus, the intergenic sequence of Family A sequences is large enough to contain a promoter and indeed there is a conserved putative −10 promoter sequence (5′-ATAATCA) and a conserved, putative Shine–Dalgarno sequence (5′-AGGC) (Figs S1 and S2) indicating a potential for a differential pattern of expression for HpuB between these two families of isolates.

Some association of sequence variants with particular *N. meningitidis* clonal complexes was evident in the phylogenetic constructions generated from the sequences of *hpuA* and *hpuB* (Figs 1 and 2). For example, distinct sequence clusters were associated with the ST-11 and ST-8 isolates with discrete clusters separating the two ST-11 electrophoretic types ET-37 and ET-15 consistent with previous studies describing this clonal complex (Jolley *et al.*, 2012). However, extensive heterogeneity was observed with evidence of lateral gene transfer among lineages.

### Detection of immune selection in the HpuAB proteins

**Fig. 3.**
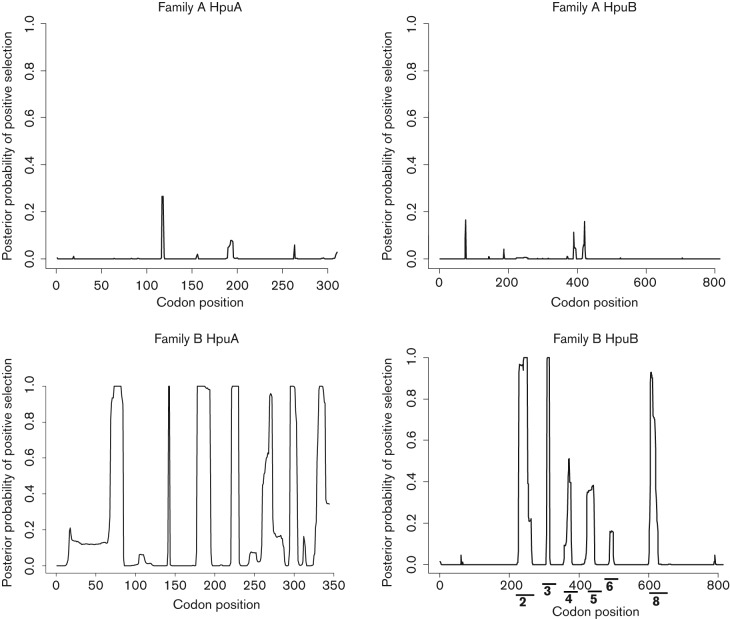
Detection of the posterior probability of positive selection acting upon Family A and B HpuAB proteins using the OMEGAMAP algorithm. Codon positions are on the *x*-axis while the *y*-axis denotes the posterior probability of positive selection. A value close to 1 is indicative of positive selection. Putative surface-exposed loops belonging to HpuB Family B proteins are denoted by black lines; the numbers indicating the loop number.

There was no evidence of immune or diversifying selection acting on Family A HpuAB sequences when analysed with the OMEGAMAP algorithm; however, posterior probability of positive selection (*d_N_/d_S_*) values >1 were detected in 74 and 51 residues in Family B HpuA and HpuB respectively (Fig. 3). Variation in the amino acid sequences encoded by these genes was localized to distinct regions of the proteins. Comparisons of the HpuA amino acid sequences from both non-pathogenic and pathogenic *Neisseria*, which ranged in length from 302 to 338 (excluding the repeat tract), detected a number of conserved residues throughout the protein, but no extensive regions of sequence identity. The majority of variation in the Family A isolates was located to two regions between residues 40–61 and 187–201 of HpuA. The variation was much more extensive in the Family B isolates with the initial region of variation extending from 16 to 85 (including an indel of 17–18 amino acids in some strains), from 177 to 195 and multiple regions in the final 125 amino acids of the protein. Comparisons of Family B HpuB amino acid sequences revealed that most of the diversity occurred between sites 217 to 267 and 599 to 629 (Table 3). These sites also contained residues with *d_N_/d_S_* values >1 and corresponded with the location of putative surface-exposed loops (Fig. 3).

**Table 3.** Diversity of HpuB regions

Region*	All	Family A†	Family B†
plug {177 aa}	0.078 (51)	0.072 (32)	0.018 (25)
t1 {10}	0.080 (2)	0.070 (2)	0.000 (0)
Loop 1 {4}	0.096 (1)	0.051 (0)	0.001 (0)
t2 {11}	0.080 (4)	0.060 (0)	0.030 (4)
i1 {3}	0.180 (2)	0.126 (0)	0.018 (2)
t3 {12}	0.060 (2)	0.040 (0)	0.010 (2)
Loop 2 {50}	0.192 (33)	0.084 (25)	0.099 (23)
t4 {16}	0.050 (3)	0.100 (3)	0.020 (0)
i2 {2}	0.276 (1)	0.120 (1)	0.252 (2)
t5 {15}	0.080 (2)	0.060 (2)	0.020 (0)
Loop 3 {23}	0.173 (13)	0.085 (10)	0.058 (7)
t6 {15}	0.100 (4)	0.070 (4)	0.020 (1)
i3 {8}	0.098 (4)	0.063 (4)	0.004 (1)
t7 {14}	0.106 (7)	0.069 (6)	0.033 (2)
Loop 4 {27}	0.131 (11)	0.104 (7)	0.061 (7)
t8 {8}	0.117 (6)	0.192 (5)	0.002 (1)
i4 {4}	0.075 (2)	0.077 (2)	0.000 (0)
t9 {22}	0.099 (8)	0.057 (7)	0.015 (1)
Loop 5 {31}	0.149 (21)	0.042 (8)	0.043 (14)
t10 {16}	0.098 (7)	0.022 (2)	0.011 (2)
i5 {4}	0.150 (4)	0.069 (4)	0.000 (0)
t11 {14}	0.079 (7)	0.095 (6)	0.007 (0)
Loop 6 {21}	0.110 (11)	0.134 (8)	0.013 (5)
t12 {14}	0.110 (5)	0.063 (5)	0.021 (1)
i6 {5}	0.156 (3)	0.105 (3)	0.000 (0)
t13 {12}	0.069 (2)	0.101 (0)	0.000 (1)
Loop 7 {26}	0.104 (4)	0.055 (1)	0.080 (3)
t14 {8}	0.104 (2)	0.049 (1)	0.064 (2)
i7 {4}	0.131 (1)	0.063 (1)	0.096 (0)
t15 {23}	0.110 (5)	0.065 (5)	0.078 (5)
Loop 8 {30}	0.132 (9)	0.090 (9)	0.109 (15)
t16 {21}	0.080 (5)	0.030 (1)	0.071 (4)
i8 {10}	0.139 (2)	0.044 (0)	0.130 (2)
t17 {13}	0.107 (4)	0.074 (2)	0.085 (3)
Loop 9 {16}	0.129 (5)	0.071 (3)	0.104 (3)
t18 {9}	0.119 (3)	0.055 (1)	0.082 (3)
i9 {5}	0.071 (2)	0.047 (1)	0.066 (1)
t19 {10}	0.101 (4)	0.099 (2)	0.026 (4)
Loop 10 {27}	0.057 (4)	0.045 (2)	0.013 (2)
t20 {11}	0.058 (3)	0.043 (3)	0.002 (1)
i10 {4}	0.096 (2)	0.065 (2)	0.000 (0)
t21 {8}	0.096 (4)	0.093 (3)	0.021 (1)
Loop 11 {41}	0.067 (8)	0.094 (6)	0.012 (3)
t22 {11}	0.064 (2)	0.124 (1)	0.007 (1)

* Regions are numbered and designated as a periplasmic loop (i), a surface-exposed loop (Loop) or a transmembrane region (t); braces show the lengths of each region in aa.

† Numbers in parentheses are non-synonymous sites. Values denote *p-*distances.

### Structural model of HpuB and location of amino acid variation

**Fig. 4.**
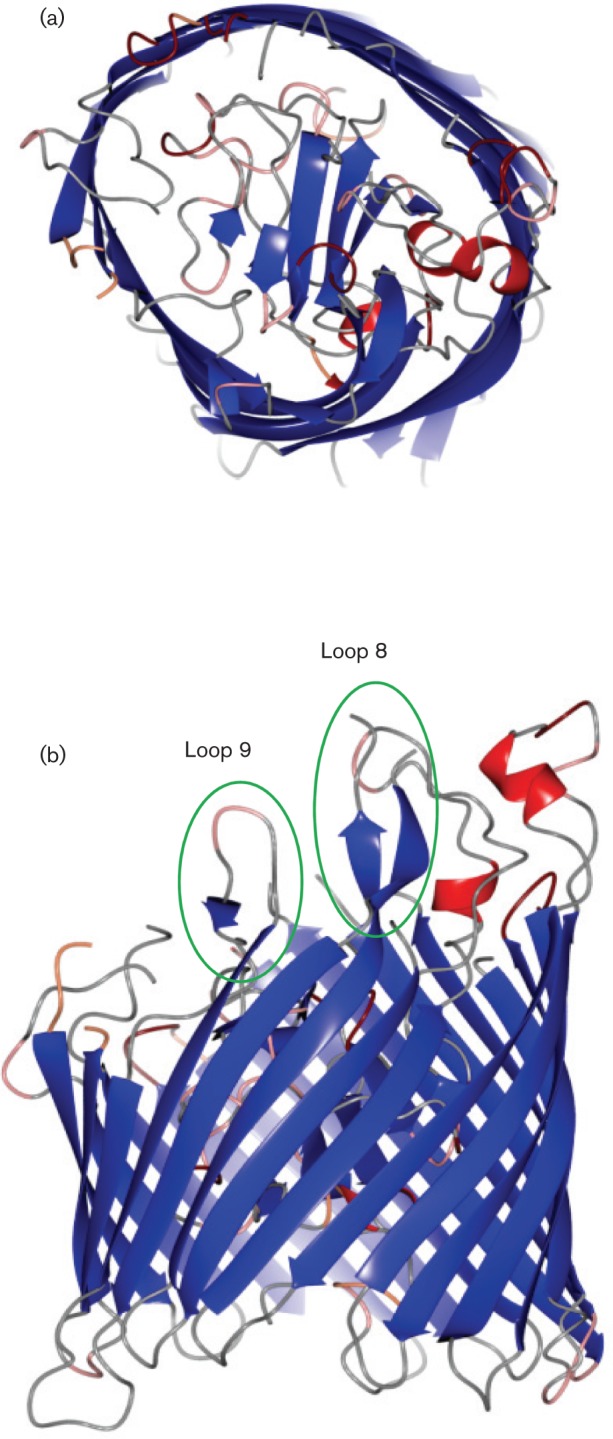
Structural model of HpuB. The structure is presented as a ribbon plot, with α-helices marked in red and β-strands in blue. (a) View from above, showing the location of the plug domain in the lumen of the β-barrel. (b) View from the side; the locations of two of the more variable sequence loops, 8 and 9, are circled and labelled.

There were no proteins with sufficiently high homology to HpuA present within the RCSB Protein database (PDB) (www.rcsb.org) for structural predictions for this protein. HpuB is a transmembrane protein and a member of the TonB-dependent transporter (TBDT) family (Noinaj *et al.*, 2010). The closest orthologue for HpuB for which a crystal structure was available was ShuA, which is a 73 kDa OMP of *Shigella dysenteriae* required for extraction of haem from haemoglobin (Cobessi *et al.*, 2010). The ShuA structure was therefore used as a template to generate a structural model for an *N. meningitidis* HpuB protein with an automated three-dimensional protein structure tool (swiss-model). The resulting structure comprised a 22-stranded β-barrel with an N-terminal ‘plug’ domain, characteristic of the TBDT family (Fig. 4a). Similarly to HmbR, it also contained 10 putative periplasmic loops and 11 surface-exposed loops (Evans *et al.*, 2010). The overall sequence homology between the HpuB and ShuA was low (16 %) and, consequently, several longer exterior loops from HpuB could not be built reliably, and were omitted from the final three-dimensional structure. The HpuB structure preserved known features common to TonB-dependent haem transporters (Fig. S3): for example, two arginine residues found in the ShuA plug domain (R64 and R104), part of the lock region (Cobessi *et al.*, 2010), were aligned with R91 and K145 in HpuB. Similarly, the ShuA FRAP/NPNL sequence, again characteristic of haem transporters (Perkins-Balding *et al.*, 2004), was aligned with a well-conserved FRAP/NPEL motif found in all HpuB sequences.

The HpuB model assisted in defining the beginnings and ends of the 22 transmembrane beta strands (Table S2). As anticipated, the predicted extra-membrane regions on the periplasmic side of the membrane (numbered 2i, 4i, etc.), were shorter than those predicted to lie on the external surface. The putative surface-exposed regions that exhibited the highest sequence variation in both families were loops 2, 8 and 9: most variability occurred in loop 2, including differences in length by up to five residues (Table 3 & Fig. S4). Loop 2 was too long to be modelled effectively, but a complete model for loop 9 was constructed and part of loop 8 (Fig. 4b). Interestingly, loop 9 was not one of the longer external loop regions although it was subject to high sequence variation (Table 3 and Table S2). This could be because sequences within the longer loop regions are constrained in their variation for functional reasons or because they fold inwards and are less exposed at the surface. In addition, loop 9 does not appear to be subject to positive selection (Fig. 3). Compared to loop 8 which has among Family B isolates 15 variable sites, loop 9 only has three which were either Lys-Thr-Gln or Thr-Asn-Lys starting at residue 674 indicating that these may be compensatory mutations.

The HpuB proteins ranged from 805 to 812 amino acids in length and exhibited higher levels of conservation between the two families compared to HpuA (Table 2). The variability of all HpuB regions was examined in Family A and Family B isolates (Table 3). The ‘plug’ domain was highly conserved in Family B isolates but showed some variation in Family A (*p-*distances 0.018 and 0.072 respectively), indicating differences in selection pressures acting on these HpuB sequences among the non-pathogenic *Neisseria* species, possibly indicative of differences in function. High levels of variation were detected in external loops 2, 3, 4, 5 and 8 in the overall analysis but loop 2 and loop 8 had the highest level of variation in Family B strains. These results were consistent with loops 2 and 8 being subject to positive selection imposed by host immune responses.

## Discussion

OMPs are considered to be among the best available candidates for a comprehensive vaccine targeting meningococci; however, these proteins are highly diverse, complicating vaccine design (Urwin *et al.*, 2004). Analysis of the diversity and selection pressure acting on OMPs is thus an important part in assessing the suitability of a given protein as a vaccine component. OMPs involved in iron acquisition are attractive vaccine candidates as iron is essential for growth *in vivo* and systemic spread of meningococci. Iron-acquisition receptors have the added benefit of potentially providing protection against both meningococci and gonococci: the recent emergence of antibiotic-resistant gonococci (Lewis, 2010), combined with an increase in gonococcal infections, indicating a need for alternative treatment and prevention strategies targeting this organism (Chisholm *et al.*, 2013; Noinaj *et al.*, 2012a). Here, an analysis of the diversity of the hapto-haemoglobin receptor HpuAB in a collection of *Neisseria* isolates was used to assess the potential of the receptor as a vaccine candidate. In so doing, information on the dynamics of iron acquisition via haemoglobin or haemoglobin–haptoglobin by *Neisseria* was generated.

While there was some clustering of *hpuAB* sequences by species, the overall lack of distinct groups was consistent with frequent movement by lateral gene transfer among members of different species. This was similar to that seen with the iron-regulated TonB-dependent enterobactin receptor FetA, also known as FrpB, which is found among both pathogenic and non-pathogenic *Neisseria* and for which a common gene pool enabling frequent genetic exchange has been described (Bennett *et al.*, 2009). Two major families, A and B of HpuAB, were identified (Figs 1 and 2). Family A *hpuAB* genes were not subject to mononucleotide repeat-mediated phase variation, which was prevalent in Family B. This suggests that additional phenotypes in haemoglobin acquisition, not based solely on the presence/absence of *hpuAB* genes, occur, including one in which *hpuAB* genes are constitutively expressed (in iron-deplete environments) and the other where *hpuAB*, specifically *hpuA*, translation can be switched on or off, the latter found in *Neisseria* associated with pathogenicity. The lack of positive selection evident in Family A HpuAB sequences was consistent with these proteins being less subject to selection pressures imposed by the immune system, which would be consistent with the absence of a need for phase variation of these variants (Fig. 3). In contrast, Family B HpuAB proteins exhibited evidence for strong diversifying selection that was consistent with immune selection acting on these variants. This was particularly apparent in HpuA sequences where 74 out of 344 amino acid residues were under positive selection as opposed to 51 out of 813 in HpuB. HpuA encodes a putative lipoprotein which is fully surface exposed when expressed, similar to the transferrin receptor, TbpB, which has been found to be particularly diverse (Rokbi *et al.*, 1997). The surface-exposed nature of these proteins has possibly generated the hyper-variability seen and, for *hpuA*, resulted in the evolution of phase variation (*tbpB* sequences containing a homopolymeric repeat have not been found to date).

HpuB is a transmembrane protein, with the positive selection signal corresponding to the putative surface-exposed loops consistent with these eliciting a bactericidal immune response, while the transmembrane, periplasmic and plug regions were more conserved (Table 3). HpuB shares approximately 28 % sequence similarity with HmbR (Fig. S3) (Lewis *et al.*, 1997) and, similarly to HmbR, the putative extracellular loops 6 and 7 identified in HpuB were conserved and had few sites under strong diversifying selection, particularly among Family B sequences (Evans *et al.*, 2010). In addition, putative loop 7 contained the conserved NPEL haem transport motif also identified in loop 7 from HmbR (Perkins-Balding *et al.*, 2004). These loops had been implicated in haemoglobin utilization in HmbR with deletion mutation resulting in reduced fitness of the organism consistent with a functional constraint limiting the diversity of these loops (Perkins-Balding *et al.*, 2003). These functional studies of HmbR also indicated that loops 2 and 3 were necessary for haemoglobin binding with mutation resulting in reduced haemoglobin-binding activity. Although these loops contained regions implicated in haemoglobin acquisition in HmbR, the association of given variable region sequences within loops 2 and 3 with particular clonal complexes indicated conservation of the haemoglobin-binding site within lineages and, combined with the detection of positively selected residues, this suggested that these surface-exposed regions contained epitopes (Evans *et al.*, 2010). Putative loop 2 from HpuB is in an equivalent position to loop 2 of HmbR and also displayed strong diversifying selection (Table 3 and Fig. 3). Some association of variable regions sequences in loop 2 with particular *N. meningitidis* clonal complexes was evident in Family B HpuB sequences (Fig. S4); however, evidence for lateral gene transfer was also apparent in this region both among meningococci and between this species and the closely related non-pathogenic species *N. polysaccharea* and *N. lactamica*, indicating that a common gene pool is associated with this receptor. In contrast, the amino acid sequence for this loop contained residues specific to *N. gonorrhoeae*, consistent with the absence of recombination in *hpuAB* between this species and other *Neisseria*.

In the related TonB-dependent receptors for transferrin and lactoferrin, the lipoproteins *tbpB* and *lbpB* are not essential and have been found to serve an accessory role by enhancing iron acquisition (Anderson *et al.*, 1994; Biswas *et al.*, 1999). In contrast, both HpuAB components are required in both gonococci and meningococci with RNA analysis demonstrating that *hpuA* and *hpuB* were co-transcribed on a single mRNA transcript (Chen *et al.*, 1998; Lewis *et al.*, 1997, 1999). Conflicting reports have, however, indicated that a 2.5 kb mRNA transcript consistent with the size of an independent mono-cistronic *hpuB* was sometimes found along with a separate report observing that point mutations in HpuB allowed gonococci to grow using haemoglobin without the expression of HpuA (Chen *et al.*, 2002; Lewis *et al.*, 1997). Eight non-polymorphisms were found in the *N. gonorrhoeae* HpuB proteins analysed in this study; however, these polymorphisms did not correlate with the previously described point mutations or with the on/off status of *hpuA* indicating that this mechanism for generating HpuA independent haemoglobin uptake is not widespread.

The distribution of the HpuAB and HmbR receptors among *Neisseria* species indicated the importance of iron acquisition from a haemoglobin source in this genus. Excluding *N. bacilliformis*, *N. weaveri*, *N. animalis*, *N. canis*, *N. dentiae* and *N. elongata,* which may inhabit diverse niches and have different nutritional requirements, all *Neisseria* species contained HpuAB alone, HmbR alone or both receptors for acquiring iron from haemoglobin. The non-pathogenic *Neisseria* species, in particular, were characterized by a high prevalence of a HpuAB only phenotype which was consistent with the observation that the haemoglobin receptor, HmbR, is a significant virulence factor in meningococcal pathogenesis (Harrison *et al.*, 2009). The prevalence of *hpuAB* genes among the closely related non-pathogenic *Neisseria* population generates a global gene pool from which extensive allelic transfer is possible, and which ultimately will limit the efficacy of these receptors as vaccine candidates (Linz *et al.*, 2000; Maiden *et al.*, 1996). Carriage of non-pathogenic *Neisseria* may contribute to the development of natural immunity to meningococcal disease, and expression of OMPs such as HpuAB and FetA/FrpB may be implicated in this immunity. The use of such cross-reactive antigens in vaccine formulations could impede the acquisition of natural immunity to meningococcal disease by preventing colonization by non-pathogenic *Neisseria* and indicates caution when designing vaccines containing cross-reactive antigens. On the other hand, the prevalence of the HmbR receptor among *N. meningitidis* isolates associated with disease combined with the absence of this receptor among non-pathogenic *Neisseria* indicates that a vaccine would be unlikely to affect the host microbiota. Furthermore, the absence of this receptor in non-pathogenic *Neisseria* narrows the gene pool available for allelic exchange thereby probably generating a more promising vaccine candidate. The presence of an HmbR pseudogene among *N. gonorrhoeae* isolates, however, indicates that this would not be a suitable vaccine candidate for this species, although it would be important to determine whether all *N. gonorrhoeae* isolates exhibit the same phenotype as the 16 isolates analysed herein.
